# What do we know about the experience of seclusion in a forensic setting? An integrative literature review

**DOI:** 10.1111/inm.13002

**Published:** 2022-04-05

**Authors:** Alison Hansen, Michael Hazelton, Robyn Rosina, Kerry Inder

**Affiliations:** ^1^ School of Nursing & Midwifery College of Health, Medicine and Wellbeing University of Newcastle Callaghan New South Wales Australia; ^2^ School of Nursing & Midwifery Faculty of Medicine, Nursing and Health Sciences Monash University Clayton Victoria Australia; ^3^ Hunter Medical Research Institute New Lambton New South Wales Australia; ^4^ Independent Researcher Sydney New South Wales Australia

**Keywords:** experience, forensic mental health, seclusion, sex

## Abstract

Seclusion is used in forensic and general mental health settings to protect a person or others from harm. However, seclusion can result in trauma‐related harm and re‐traumatization with little known about the experience of seclusion for consumers in forensic mental health settings from their perspectives. This article explores consumer experiences of seclusion in forensic mental health settings and explores the differences between female and male experiences of seclusion. Five electronic databases were systematically searched using keywords and variations of experience, attitude, seclusion, coercion, forensic mental health, and forensic psychiatry. Inclusion criteria were original peer‐reviewed studies conducted in adult forensic mental health settings reporting data on the experiences of or attitudes towards seclusion. Seven studies met the criteria for inclusion and a quality assessment was undertaken. Results found consumers in forensic mental health settings perceive seclusion to be harmful, a punishment for their behaviour, and largely a negative experience that impacts their emotional health. Some consumers report positive experiences of seclusion. Differences in the experience of seclusion for females and males are unclear. Further research is required to understand the experience of seclusion for women in forensic mental health settings. Identification and consideration of differences in the experience of seclusion for males and females may assist in identifying sex‐specific interventions and may inform policy and practices to eliminate or reduce the trauma associated with seclusion use.

## INTRODUCTION

Seclusion reduction and eradication are a priority in mental health settings globally and in Australia (Barr *et al*. 2019). The Royal Commission on Victoria's Mental Health System has recommended immediate action to reduce the use of seclusion with the aim of eliminating this practice in 10 years (State of Victoria 2021). The use of seclusion in mental health settings is controversial (Long *et al*. 2015). Seclusion use infringes on human rights and is associated with a range of risks for consumers who experience seclusion (Huckshorn 2006). Seclusion has been identified as a type of avoidable harm (Newton *et al*. 2017) and continues to be used without evidence for its benefit to the person being secluded, or evidence of treatment effectiveness (Turner & Mooney 2016). The harm experienced by consumers who are secluded can be physical, psychological, and may cause distress (Chieze *et al*. 2019; Melbourne Social Equity Institute 2014), with negative experiences of seclusion persisting following the cessation of seclusion (Meehan *et al*. 2000). Contemporary mental health practice aims to support consumer recovery and recognize and reduce the impact and exacerbation of trauma. However, trauma occurs as a result of the use of seclusion, with seclusion related trauma impacting recovery (Brophy *et al*. 2016b), highlighting ‘the adverse effects of seclusion …are incompatible with recovery and trauma‐informed care and practice’ (Melbourne Social Equity Institute 2014, p. 17).

Most research conducted on seclusion has taken place within general mental health settings. Several studies exploring the consumer experience of seclusion in general mental health services (e.g. Kinner *et al*. 2017; Larue *et al*. 2013; Mayers *et al*. 2010; Meehan *et al*. 2000) report that consumers have mixed feelings towards seclusion (Larue *et al*. 2013), from beneficial to increase safety, to being harmful (e.g. Kinner *et al*. 2017). The use of seclusion is largely perceived as a negative experience and associated with causing harm and distress (Kinner *et al*. 2017; Mayers *et al*. 2010); persisting long after the seclusion event has ceased (Meehan *et al*. 2000).

Consumers who have experienced seclusion in general mental health settings report its use as punitive (Mayers *et al*. 2010) or a punishment for their behaviour, with some expressing that seclusion had been used inappropriately (Meehan *et al*. 2000). Disempowerment and powerlessness have been reported to be related to feelings of humiliation and a sense of fear (Meehan *et al*. 2000). Seclusion has been associated with feelings of a loss of dignity and a violation of human rights (Mayers *et al*. 2010) and, can exacerbate existing trauma (Kinner *et al*. 2017). International and national bodies stipulate that seclusion should not be used as a punishment, or for staff convenience, or as a form of discipline (Melbourne Social Equity Institute 2014). Despite this, previous research indicates consumers who have been secluded do feel seclusion is used in these ways (Larue *et al*. 2013; Meehan *et al*. 2000).

Research conducted on seclusion within general mental health settings may not be reflective of the experiences of seclusion for consumers in forensic mental health settings. While general mental health settings and forensic mental health settings both support people in their recovery, there are key differences between these types of service settings. The key differentiating feature is that forensic mental health settings provide a safe and secure environment for consumers who have a mental illness and whose behaviour has led to or, has the potential to lead to, offending behaviour (Mullen *et al*. 2000), or consumers admitted from general mental health settings who require a more secure environment.

The physical environment between forensic and general settings also differs to provide a secure environment. Forensic mental health settings are restrictive with high levels of security (Tomlin *et al*. 2019) to manage risk ‘on account of dangerousness’ (Keski‐Valkama *et al*. 2010, p. 447). Admission to a forensic mental health setting may be considered to be coercive in itself, as users are not admitted voluntarily (Lau *et al*. 2020). While admission is justified on account of managing public safety (Lau *et al*. 2020) and supporting mental health needs, the nature of the forensic environment may further exacerbate trauma associated with restrictive practices, with forensic settings having been found to be more stressful than non‐forensic settings (Soininen *et al*. 2016).

The use and duration of coercive practices including seclusion differs between types of services (Lepping *et al*. 2016; Steinert *et al*. 2010) and between different countries (Al‐Maraira & Hayajneh 2019). Seclusion is used more frequently and for longer durations in forensic mental health settings compared to general mental health settings (Flammer *et al*. 2020; Maguire *et al*. 2021) with further variation across different forensic settings (Hui *et al*. 2013). The reasons for these differences are unclear, however, differences in demographics and complexity of presentations, and differences in service approaches to care delivery, assessment and management of risk, and recovery, staff culture (Newton *et al*. 2017), local practice (Maguire *et al*. 2021), and attitudes towards seclusion may contribute (Bowers *et al*. 2007). There may be a greater risk of harm and seclusion‐related trauma for consumers in forensic mental health settings due to the higher rates of seclusion use and long duration of seclusion episodes (Australian Institute of Health & Welfare 2021).

Globally, forensic mental health services have seen increases in demand, and subsequent increases in bed capacity and resources (Jansman‐Hart *et al*. 2011). While the vast majority of forensic mental health service users are male, research suggests that over the past two decades there have been significant increase in the number of females admitted to forensic mental health services (de Vogel & Nicholls 2016). This is reflective of the rapid rise in female incarceration at higher rates than males (e.g. Fedock *et al*. 2013).

Females in forensic mental health settings have unique experiences in relation to their psychosocial, clinical, and criminological presentation compared to males (Nicholls *et al*. 2015). Females typically have more extensive histories involving trauma and abuse (e.g. Bartlett *et al*. 2014; Cooke & Bailey 2011) and while admitted are involved in more frequent incidents of violence (Parkes & Freshwater 2012) and self‐harm (de Vogel & Nicholls 2016) than males. The use of seclusion therefore may exacerbate existing trauma and cause significant harm to a population already highly vulnerable.

Given the increase in demand for forensic mental health services and the need to support seclusion reduction and eradication, a synthesis of current knowledge and understanding of the experience of seclusion in this setting will allow clinicians to identify and reflect on key practice issues in a timely and succinct way.

### Aims

This integrative literature review aims to synthesize published research on the experience of or attitude towards seclusion for consumers in forensic mental health services, and to explore whether there are differences in the experience of seclusion for females and males.

## METHODS

An integrative review using a systematic approach was conducted to explore published literature pertaining to the experience of seclusion for females and males in forensic mental health settings. An integrative review was chosen as it allows for the inclusion of varying research methodologies, theoretical knowledge, and has a greater potential to contribute to theory development and evidence‐based nursing practice (Whittemore & Knafl 2005). The framework proposed by Whittemore and Knafl (2005) was utilized for this review and includes the following stages: identification of the problem, literature search, evaluation and analysis of data, and presentation. The problem identification stage of the framework describes the need for the problem that the review aims to address, to be clearly articulated (Whittemore & Knafl 2005); the aim of this review has been described above. Stage two includes the literature search strategy and stage three includes data evaluation to assess the overall quality of included data (Whittemore & Knafl 2005); this is described below. Stage four includes describing the analysis of data.

Electronic databases searched in August 2021 included CINAHL, PsycINFO (Ovid), Medline (Ovid), EMBASE, and Web of Science. An internet search to identify grey literature was conducted using the Google Scholar search engine where the first 200 hits were reviewed as recommended by Haddaway *et al*. (2015). Keywords used separately and in combination in the searches were experience*, attitude*, seclusion, restrict*, coerci*, forensic psychiatry, and forensic mental health. Papers published from 2000 onwards were included to take account of contemporary mental health nursing practice which is recovery‐focused and generally reflective of the need to reduce restrictive practices. Inclusion criteria were: original peer‐reviewed research studies conducted in forensic mental health settings for adults reporting data on the experience of or attitude towards seclusion or grey literature reporting on the experience of seclusion for consumers in forensic mental health settings. Papers were excluded if the research was conducted in general mental health settings or described nurses' experiences of seclusion use.

The relevant Joanna Briggs Institute (JBI) critical appraisal tool was used to assess the quality of the research (Lockwood *et al*. 2015; Moola *et al*. 2017) depending on the study design. The quality analysis assessed the study purpose, relevant background literature, study design, sampling, data collection and analysis, outcomes, intervention, overall rigour, conclusion, and clinical implication. Inclusion criteria were clearly documented and applied consistently to reduce the risk of potential bias (McDonagh *et al*. 2013).

Data were extracted from included papers and related themes were identified and synthesized. The first author (AH) extracted all data through the identification of themes; the subtitles in the findings section are reflective of the themes identified. The last author (KI) then checked for inconsistencies to ensure an unbiased and thorough interpretation of the data (Whittemore & Knafl 2005). The results of data extraction are described in the findings section of this review and presented in Table 1.

**Table 1:** Summary of literature reviewed on the experience of seclusion in a forensic mental health setting

Author (year) Country	Study design and setting	Aim/s	Sample size	Data sources	Key findings
Askew *et al*. (2019) England	Interpretative phenomenological analysis; medium secure hospital (rehabilitation ward and assessment ward)	To understand the individual personal experience of seclusion	*n* = 7 Males *n* = 7	Semi‐structured interviews with patients who had experienced seclusion (seclusion had to be experienced 28 days or more prior to the interview)	Four themes were identified: Intense fear Not getting the care I needed I am being abused Power struggle
Haw *et al*. (2011) United Kingdom	Retrospective cohort study; Forensic rehabilitation wards (low and medium secure wards and one open ward)	To report patient’s experiences and preferences for seclusion, physical restraint, and forced medication	*n* = 57 Females *n* = 30 Males *n* = 27	Semi‐structured interviews with patients who experienced two of the following in the last 2 years: seclusion, physical restraint, and emergency intramuscular medication	Themes identified and the number of responses related to the theme: A quiet time for reflection (*n* = 56) Prevents violence to self and others (*n* = 41) Unpleasant physical environment (*n* = 29) Unpleasant thoughts and emotions (*n* = 114) Physical pain, injury, fear of death (*n* = 85) Control (*n* = 24) Loss of privileges (*n* = 9) Indifference (*n* = 13) Positive attitudes and experience of staff conducting coercive treatments (*n* = 8) Negative attitudes and experience of staff conducting coercive treatments (*n* = 52) Coercive treatment as a positive (*n* = 9), negative (*n* = 31) or neutral (*n* = 8) experience Seclusion as both a positive and negative experience (*n* = 7)
Holmes *et al*. (2015) Canada	Modified interpretative phenomenological analysis; forensic psychiatric hospital	To explore the lived experience of the seclusion room	*n* = 13 Sex not stated	Semi‐structured interviews with patients who experience seclusion in the 6 months before the interview	Three themes were identified: Experiencing seclusion: responses varied from positive to negative experiences. Assessing the quality of care: responses included attention from nursing staff, attitudes towards nurses who secluded them, and differences between being in/out of seclusion. Space of confinement: responses related to physical space, including the level of comfort, privacy, and how this was experienced.
Hui (2017) United Kingdom	Qualitative study; high secure forensic hospital	To explore patients’ experiences of restrictive practices and interventions	*n* = 28 Females *n* = 9 Males *n* = 19	Semi‐structured interviews with patients	Three core themes were identified: Patient experiences of the high‐security hospital environment Experiences of restrictive practices and interventions Working towards overcoming trauma and adversity Seclusion is viewed as negative, a punishment, the removal of clothing particularly upsetting.
Keski‐Valkama *et al*. (2010) Finland	Retrospective cohort study; Two forensic psychiatric hospitals and two general psychiatric in‐patient units	To determine (1) knowledge of reasons for seclusion, and whether self‐reported reasons corresponded to patient files, (2) whether the patients regarded seclusion as positive or negative or both, (3) patients perceptions of interaction with staff during seclusion, and (4) any suggested improvements on the use of seclusion	Baseline: *n* = 106 Forensic *n* = 68 (males 76.5%) Follow up: *n* = 83 (78.3%) Forensic 69.9%	Structured Interview with patients who experienced seclusion shortly after seclusion ended (median time 6 days), with follow‐up interview half a year later	Seclusion as: Positive *n* = 11 (19.3%) Negative *n* = 34 (59.6%) Both *n* = 12 (21.1%) Beneficial *n* = 31 (54.4%) Harmful *n* = 12 (21.1%) Both *n* = 14 (24.6%) Punishment *n* = 49 (73.1%) Not punishment *n* = 18 (26.9%) Visits during seclusion: Sufficient *n* = 37 (56.9%) Insufficient *n* = 20 (30.8%) Indifferent *n* = 8 (12.3%) Discussions during seclusion: Sufficient *n* = 24 (36.9%) Insufficient *n* = 27 (41.5%) Indifferent *n* = 14 (21.5%) Seclusion necessary: Yes *n* = 58 (89.2%) No *n* = 7 (10.8%)
Pulsford *et al*. (2013) United Kingdom	Cross‐sectional study; High secure hospital	To ascertain and compare beliefs of staff and patients as to the causes of and best means of responding to aggressive and violent incidents	*n* = 26 Females *n* = 0 Males *n* = 23 Not stated *n* = 3	An adapted version of the Management of Aggression and Violence Attitude Scale (MAVAS)	Patient responses to the MAVAS statements regarding the use of seclusion: When a patient is a violent seclusion is one of the most effective approaches *n* = 26 (mean 3.30, agree) Practice of secluding violent patients should be discontinued *n* = 26 (mean 2.53, disagree) Seclusion is sometimes used more than necessary *n* = 26 (mean 2.42, disagree)
Tomlin *et al*. (2019)	Mixed methods (qualitative interview method reported in the article); Secure forensic hospitals (low, medium, and high)	To explore patients’ experiences of the restrictiveness of forensic mental health services	*n* = 18 Females *n* = 2 Males *n* = 16	Two mini focus groups (*n* = 2 and *n* = 3) and semi‐structured interviews (*n* = 13)	Five global themes and 21 organizing themes were identified and depicted as a Model of Restrictiveness. Seclusion was described by participants as punishing, rare, boring, or distressing to witness

In this review, the term consumer has been used to describe people admitted to a forensic mental health service, however, where included studies described participants as patients, the term patient has been retained to accurately reflect and stay true to the authors’ data. For the purpose of this paper, sex has been chosen for reporting where included papers referred to males and females, acknowledging that sex describes the biological characteristics of being male or female, whereas gender describes the socially constructed roles and expectations of males and females (Phillips 2005).

## RESULTS

**Fig. 1:**
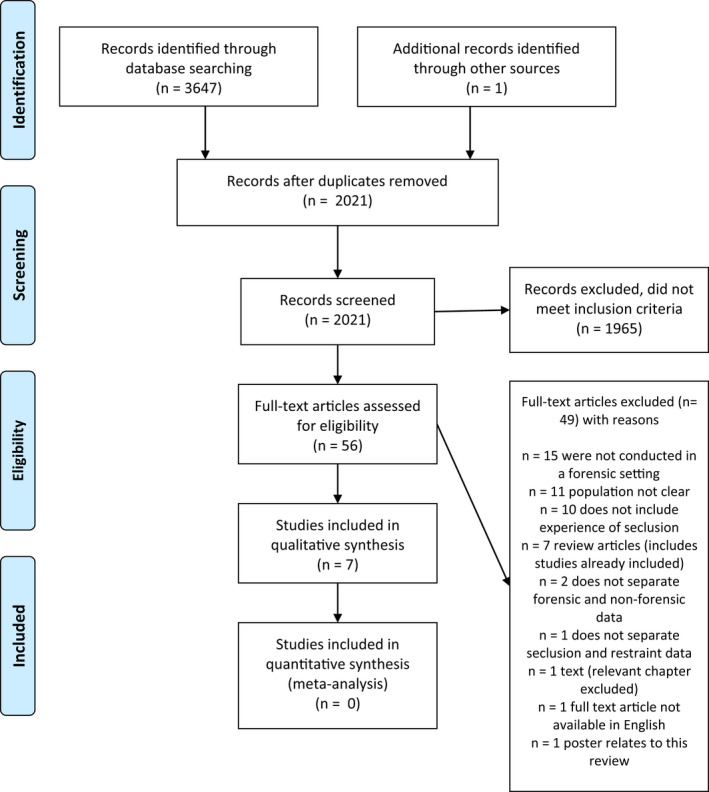
PRISMA 2009 flow diagram.

The database search returned 3647 results. One article was further identified through other sources. After de‐duplication, 2021 titles and abstracts were screened for relevance by the first author (AH). A total of 56 full‐text papers were selected and assessed for eligibility by one author (AH) using clear inclusion and exclusion criteria determined by all authors. Of these, 49 were excluded as they did not meet inclusion criteria due to being conducted in general mental health settings, or the population was not identified as a forensic population, or forensic data were not reported separately to non‐forensic data, or were a poster presentation or a review which contained original papers already included. A total of seven studies met the inclusion criteria and all met criteria for quality. All studies demonstrated high‐quality design and robust methodology determined through quality assessment using the relevant JBI critical appraisal tool (Lockwood *et al*. 2015; Moola *et al*. 2017). Figure 1 depicts the search process and results. The Google internet search returned approximately 2420 results. The first 200 results were screened, however, no additional papers met the inclusion criteria.

Of the seven papers included, reference lists were searched by hand to identify additional papers. Eight additional papers were identified however on review did not meet inclusion criteria. Characteristics of the seven studies included in this review are summarized in Table 1. As the aim of this review was to explore consumer experiences of or attitudes towards seclusion, not all data from the included studies have been summarized in the table.

### Overview of studies included in the literature review

The included studies were two retrospective cohort studies (Haw *et al*. 2011; Keski‐Valkama *et al*. 2010), a mixed‐methods study (qualitative phase reported in the published study; Tomlin *et al*. 2019), one cross‐sectional study (Pulsford *et al*. 2013), an interpretative phenomenological analysis (Askew *et al*. 2019), a phenomenological study (modified interpretative phenomenological analysis; Holmes *et al*. 2015), and a qualitative study using narrative inquiry (Hui 2017). The studies were conducted in the United Kingdom, Canada, and Finland in forensic psychiatric hospitals and forensic rehabilitation wards (low and medium secure wards and one open ward). Some studies reported data outside the scope of this review, however, were included as these did report data relevant to the aim of this review. One study included experiences of, and preferences for, physical restraint, forced medication, and seclusion (Haw *et al*. 2011); only the experience of seclusion was included in the review. Another study explored restrictive interventions and restrictive practices within forensic settings; only the experience or attitude towards seclusion was included in the review (Hui 2017). One study included both patients and nurses (Holmes *et al*. 2015); only the experiences of patients were included in the review. One study compared views of seclusion for patients in forensic and general mental health settings (Keski‐Valkama *et al*. 2010); only the experiences and views of forensic patients were included in the review. Finally, the study by Pulsford *et al*. (2013) used an adapted version of a tool that examined beliefs related to the management of aggression and violence; only specific data pertaining to the use or experience of seclusion were included in the review. Positive and negative experiences of and attitudes towards seclusion were reported in the included studies. The experiences of and attitudes towards seclusion for males and females in forensic mental health settings were identified and are discussed.

### Negative experience of seclusion

Four studies included in this review reflect that participants perceived seclusion as a negative or harmful experience (Haw *et al*. 2011; Holmes *et al*. 2015; Hui 2017; Keski‐Valkama *et al*. 2010). Some participants felt seclusion used to be stigmatizing (Keski‐Valkama *et al*. 2010), others felt intense fear (Askew *et al*. 2019), were fearful of losing control (Haw *et al*. 2011), feared being secluded again (Keski‐Valkama *et al*. 2010), or were bored and lonely (Holmes *et al*. 2015).

The use of seclusion was perceived to be a punishment (Haw *et al*. 2011; Hui 2017; Keski‐Valkama *et al*. 2010), with forensic patients perceiving its use as punishment more often than general psychiatric patients (73.1% compared to 54.1%, respectively; Keski‐Valkama *et al*. 2010). Participants who described seclusion as a punishment, felt that its use as a consequence of bad behaviour, the reason for seclusion was unknown, or the setting was inhumane and lonely (Keski‐Valkama *et al*. 2010). Similarly, Tomlin *et al*. (2019) found that participants described seclusion as punishing, however, the authors note that coercive measures were not discussed at length during interviews. Holmes *et al*. (2015) found that participants felt seclusion was misused or overused by staff. ‘I think at times it may get misused a little bit…they could be interacting with that person and helping them, rather than using the seclusion room’ (Holmes *et al*. 2015, p. 205).

Studies report other impacts of seclusion use, in relation to how seclusion may impact occupations and activities. Keski‐Valkama *et al*. (2010) found seclusion use was perceived as harmful as it contributed to a loss of ‘acquired permissions’ (11.5%) (p. 453); in the context of this study this could be assumed to mean access to something or to do something. However, the number of participants from the forensic population group that felt this way is unclear. Similarly, Haw *et al*. (2011) found descriptions of losses related to loss of privileges and control. Holmes *et al*. (2015) found descriptions of missing out on experiences while in seclusion and Hui (2017) found a lack of occupation and control contributing to the austerity of the seclusion room.

Negative or harmful experiences of seclusion were also expressed and described as emotional responses. Feelings of fear, shame, anger, and loneliness were reported (Haw *et al*. 2011; Keski‐Valkama *et al*. 2010). One participant stated ‘I get really scared by it’, another stated ‘it brings on intense feelings of shame, embarrassment and humiliation’ (Haw *et al*. 2011, p. 575). For participants who had experienced sexual abuse in the past, seclusion was difficult when being required to wear a rip‐proof gown; ‘when they strip you off even if you have a history of self‐harm they will strip you off. If you have had sexual abuse this is not very good’ (Haw *et al*. 2011, p. 575). Similarly, Hui (2017) reported participants found the removal of clothing upsetting, with a participant describing the experience of seclusion as bringing back past memories. Participants in the study by Keski‐Valkama *et al*. (2010) described harmful experiences of seclusion related to its use negatively affecting their psychiatric condition (38.5%), experiences of stigmatization or ostracisation (34.6%) and fear related to being re‐secluded (3.9%); the percentage of the forensic population group that felt this way was not reported.

Hui (2017) reported participants’ experiences of observing restrictive interventions being used on other people, with these observations portraying seclusion use in a negative light. Participants described feelings of empathy and pity for the person being secluded (Hui 2017). Seclusion was also described as being ‘disturbing…just seeing it, it’s a bit disturbing’ (p. 10). Another implied issue related to human rights, ‘…what you doing that’s a human being there’ (p. 10) and described that seclusion should not be used in hospitals.

### Positive experiences of seclusion

Three studies included in this review reported participants describing positive experiences of seclusion; two were forensic psychiatric hospitals (Holmes *et al*. 2015; Keski‐Valkama *et al*. 2010) and the other was a rehabilitation ward (Haw *et al*. 2011). This implies that despite its overall negative association, some participants who were secluded did see some benefit from its use. Some report that seclusion allows time to reflect on what happened and to learn a lesson from the experience (Haw *et al*. 2011; Keski‐Valkama *et al*. 2010). Keski‐Valkama *et al*. (2010) found similar positive experiences of seclusion, with participants reporting that seclusion was partly beneficial and allowed them to learn to control their behaviour, had a positive effect on their psychiatric diagnosis, and allowed for privacy; however, the authors did not elaborate as to why participants felt this way. Keski‐Valkama *et al*. (2010) found that forensic patients reported more beneficial than harmful effects of seclusion (54.4% vs 21.1%).

Other positive themes, similar to seclusion allowing a sense of control, including the notion of helpful isolation, allowing space away from others that enables time to calm down (Haw *et al*. 2011). In another study, one participant stated ‘I was kind of happy to be in there. I knew I didn’t have to worry about anything’ (Holmes *et al*. 2015, p. 204). Holmes *et al*. (2015) found that some participants understood the need for seclusion rooms in a forensic psychiatric setting, describing seclusion as a necessity and a tool that can keep people safe.

### The physical environment (seclusion room)

Largely, the physical environment of the seclusion room is experienced as being an uncomfortable, horrible, and unpleasant space (Haw *et al*. 2011; Holmes *et al*. 2015), likened to being in prison (Haw *et al*. 2011) and a confined space (Holmes *et al*. 2015). The seclusion space was also described as boring and demoralizing (Holmes *et al*. 2015) and claustrophobic (Hui 2017).

Askew *et al*. (2019) found that the physical aspects of the seclusion experience were associated with fear in relation to staff and the environment. One participant described being fearful when and how staff entered the seclusion room (in response to hiding and covering of the observation panel) ‘…every time they open the door, they kinda like in gloves and there was about 12 of them, I thought, what the fuck’s going on here…ideas in my head thinking they’re gonna fuckin’ kill me’ (Askew *et al*. 2019, p. 5).

The physical environment of seclusion was also reported to provide sanctuary. However, it is unclear whether the physical seclusion room was perceived to be a sanctuary (Haw *et al*. 2011), or whether the seclusion provides a sense of sanctuary, in that it provides a sense of safety or comfort. Further, seclusion was described as being boring (Tomlin *et al*. 2019), however participant elaboration on this was not discussed, and again the authors note that coercive measures were not discussed at length during interviews.

### Differences in the experience of seclusion for females and males

Not all studies included females and males in their sample or separated female and male data. Two of the six studies (Askew *et al*. 2019; Pulsford *et al*. 2013) did not include female participants in their sample and one study (Holmes *et al*. 2015) did not identify the sex of the participants. Four studies (Haw *et al*. 2011; Hui 2017; Keski‐Valkama *et al*. 2010; Tomlin *et al*. 2019) included male and female participants in their sample (refer to summary Table 1) with the majority being male. The study by Haw *et al*. (2011) had the largest sample of women (*n* = 30). None of the studies that included both sexes reported the participant’s experiences of seclusion by their biological sex. This may be due to there being a smaller number of females than males in forensic populations (Long *et al*. 2008; Ribeiro *et al*. 2015).

Haw *et al*. (2011) identified female experiences of seclusion in two contexts. The preservation of dignity was a suggestion for seclusion use to be more acceptable, for example, the consideration of privacy when clothing and disrobing were required to support dignity. A female participant described coercive interventions affecting their leave, privileges, and progress towards discharge, stating ‘You’ve got to start again, lose leave, lose this, lose that’ (Haw *et al*. 2011, p. 576). Whether this response was in relation to the experience of seclusion, or another coercive intervention (physical restraint or forced medication) explored in the study is unclear.

### Staff actions and interactions while in seclusion

The studies reflected a range of perceptions in relation to staff interaction, received attention, and quality of care during seclusion. Experiences ranged from staff being perceived to be caring, skilled, and supportive to staff being perceived in a negative light (Haw *et al*. 2011) or hard to interact with (Hui 2017), to patients feeling neglected or abused (Askew *et al*. 2019). Keski‐Valkama *et al*. (2010) found that some participants reported feelings of indifference towards staff interaction, which were interpreted to reflect participants’ cynical attitudes derived from seclusion.

One study reported that consumers felt they received less attention in seclusion (than when not in seclusion), and attitudes towards nursing staff who were secluded were clearly negative (Holmes *et al*. 2015). ‘The staff don’t come to you when you need help’; ‘They keep you in there too long sometimes. They don’t really talk to you, they don’t care’ and one participant stated ‘[nurses] are just as capable of violence as the patients are’ (Holmes *et al*. 2015, p. 205). Similarly, feelings of neglect and abandonment were identified, which challenged the expectation of care (Askew *et al*. 2019). One participant described being ‘left (by staff)’ and another stated ‘…there is no help. It’s just feels totally like, abandoned, helpless…’ (Askew *et al*. 2019, p. 5).

Staff actions were also interpreted as a form of abuse, from physical to sexual abuse which were also discussed alongside feeling fearful (Askew *et al*. 2019). This occurred in the context of staff observing behaviour while in seclusion or in the bathroom or shower, when they were required to enter seclusion or when items needed to be removed from seclusion to maintain safety (Askew *et al*. 2019). The authors note, while study participants did not make allegations of abuse occurring, staff actions were interpreted as abusive; participants described that they felt like victims of staff abuse (Askew *et al*. 2019). Further, staff behaviour was seen as deliberate neglect (Askew *et al*. 2019).

The study by Askew *et al*. (2019) found that seclusion resulted in experiences of loss and gain of power, with some participants describing feelings of powerlessness during seclusion, while others sought out ways to gain power. Participants described staff having control over the duration and experience of their seclusion, resulting in participants being passive for seclusion to cease (Askew *et al*. 2019). One participant described seeking power (due to feeling powerless) by refusing to leave the room or openly masturbating. Another assessed staff, their qualifications and their abilities as a response to his risk being assessed, and felt the long duration of his seclusion was a result of a lack of training (Askew *et al*. 2019).

### Suggestions for seclusion use

The majority of studies included participant’s opinions about future seclusion use or suggestions for seclusion use (Haw *et al*. 2011; Holmes *et al*. 2015; Keski‐Valkama *et al*. 2010). Participants’ responses in one study varied from never wanting to be secluded again to wanting to be able to request seclusion (Haw *et al*. 2011). To make the seclusion process better, some participants recommended being able to walk into seclusion by themselves, the use of de‐escalation techniques, a softer physical environment in seclusion, and a clear explanation of why seclusion was being used (Haw *et al*. 2011). Female participants wanted their dignity preserved and not to have their clothing removed (Haw *et al*. 2011). Although Holmes *et al*. (2015) did not explicitly ask participants about ways to improve seclusion, suggestions included that cameras may make seclusion safer, while also allowing nurses to see how the person is coping and whether they may be ready to be released from seclusion (Holmes *et al*. 2015).

Approximately half of the patients in the study by Keski‐Valkama *et al*. (2010) suggested at least one alternative that would have helped at the time of their seclusion event. These included resting in their own room (51.9%), verbal de‐escalation (46.3%), use of medication (40.7%), and activities (18.5%). The authors report no statistically significant differences in the opinions of the forensic and general psychiatric participant groups (Keski‐Valkama *et al*. 2010), however, alternatives proposed by which participant group were not clear.

While suggestions for future seclusion use were not discussed explicitly, interestingly and in contrast to previous literature and approaches to reduce and where possible eliminate the use of seclusion, Pulsford *et al*. (2013) found that participants disagreed with the discontinuation of seclusion for violent patients. Staff disagreed more than patients (mean 1.48 and 2.53, respectively) and participants regarded seclusion as valuable.

### Limitations of included studies

Despite all papers included in this review being assessed for quality using the relevant Joanna Briggs Institute (JBI) critical appraisal tool (Lockwood *et al*. 2015; Moola *et al*. 2017), studies included in this review had a number of methodological limitations. Two studies (Askew *et al*. 2019; Haw *et al*. 2011) were conducted in forensic rehabilitation wards, which included low and medium secure wards, an open ward, and an assessment ward. Therefore, the findings from this study may not be generalizable to high secure forensic wards, as consumers secluded in wards or hospitals with high levels of security may experience seclusion differently. Similarly, two other studies included in the review (Holmes *et al*. 2015; Keski‐Valkama *et al*. 2010) identified the service setting as forensic psychiatric hospitals, however, the level of security was not identified. The clear identification of the service type and security level was identified during the assessment of quality (Lockwood *et al*. 2015; Moola *et al*. 2017) however, did not impact inclusion in this review. Conversely, findings from studies conducted in high secure settings (Pulsford *et al*. 2013) may not be generalizable to low or medium secure settings. The experience of seclusion may differ for consumers depending on the level of security, warranting further investigation.

The study by Haw *et al*. (2011) included only patients who had experienced two types of coercive treatments (seclusion, physical restraint, or emergency intramuscular medication) in the past 2 years, which may have impacted on eligibility for inclusion and may not be representative of consumers who had experienced seclusion only. The authors reported that some participants experienced difficulty in recalling coercive treatment over the past 2 years (Haw *et al*. 2011). Themes about participants’ experiences of coercive treatments, related to which specific type of coercive treatment was unclear. Further, quantitative data on the number of participants who identified themes of coercive interventions were not separated by the coercive intervention (Haw *et al*. 2011). This restricts the ability of the authors to comment on specific themes relating to the experience of seclusion. Additionally, as identified by the authors, all authors included in the study (Haw *et al*. 2011) were employed or had been employed by the service. This ethical issue was considered and managed by authors not interviewing participants who had previously been involved in their care (Haw *et al*. 2011) and was noted during the quality assessment (Lockwood *et al*. 2015).

The study by Keski‐Valkama *et al*. (2010) examined forensic and general psychiatric patients’ views of seclusion and did not report all data for each population group separately. However, the authors report that forensic patients and general patients do experience seclusion in a similar way, except forensic patients perceive seclusion to be more of a punishment. This is an area that requires further investigation. Keski‐Valkama *et al*. (2010) noted that qualitative data were too sparse for analysis, highlighting the need for further research to understand the experience of seclusion in forensic mental health settings. A larger study across similar services may assist in being able to develop an understanding of the experience of seclusion for consumers in forensic mental health settings and allow for benchmarking across services. Findings may provide evidence to reduce or eliminate the use of seclusion and or reduce the trauma associated with seclusion use.

None of the studies separated all responses by the consumer’s sex, which does not allow the second aim of this review, to determine whether there are differences in the experience of seclusion for females and males, to be met. This limits the voice of female consumers being adequately represented (Lockwood *et al*. 2015) and would have increased overall quality through clearly acknowledging and reporting sex. The lack of the experience of seclusion for women being clearly described highlights the need for further research to explore whether there are differences in how males and females experience seclusion, particularly in the context of differences identified within this population prior to and during admission.

Finally, there were no data identifying participants who identify as non‐binary, and to the authors’ knowledge, there is no published literature pertaining to the experience of seclusion in forensic mental health settings for people who identify as non‐binary gender. This is a future area of research in the context of ensuring care is gender‐sensitive, as gender differences can impact mental health, expression and experience of mental health problems, and treatment needs (Judd *et al*. 2009).

## DISCUSSION

This integrative review explored and synthesized the key themes from published research on the experience of seclusion for consumers in forensic mental health services and explored differences between female and male experiences of seclusion. A total of seven original research papers met inclusion criteria. Findings suggest that seclusion is largely perceived as a negative experience, harmful, and punishment for behaviour, negatively impacting the emotional health of those subjected to it. Few studies have examined the experience of seclusion for consumers in forensic mental health settings or examined differences between male and female experiences of seclusion, despite documented differences between males and females within this setting. A succinct report of the findings related to the experience of seclusion use for consumers in forensic settings, allows clinicians to reflect on the potential impact of seclusion use, and may support consideration of practice related to seclusion reduction and eradication.

This review found that consumers in forensic mental health settings perceive seclusion as a punishment more often than consumers in general mental health settings (Keski‐Valkama *et al*. 2010). The reason for this, and whether consumers of forensic mental health settings perceive this more generally is unclear (Hui *et al*. 2013). A number of factors could influence the differences between the experience of seclusion for general and forensic consumers. For example, differences between the consumer populations, current and past experiences related to trauma, and the physical environment, have been found to be more stressful than in non‐forensic settings (Soininen *et al*. 2016). As earlier described, these differences may exacerbate existing trauma and increase the risk of re‐traumatization when seclusion is used to manage behaviour. Therefore, consideration should be paid to the impact the highly secure environment may have on experiences of seclusion. While seclusion was largely perceived to be a negative experience, there are data indicating that there are also positive experiences of seclusion. This finding should be interpreted with caution, as reporting a positive experience of seclusion does not indicate that the experience of seclusion is beneficial, however, positive experiences suggest potential issues related to the physical environment and lack of space or privacy in forensic mental health settings.

This review was unable to determine whether male and female experiences of seclusion differ. This is an identified limitation as females in forensic mental health settings have unique and different experiences in relation to their presentation compared to males (Nicholls *et al*. 2015). While females are a minority in forensic settings (Ribeiro *et al*. 2015; de Vogel & Nicholls 2016), studies suggest an increase in the number of females being managed in forensic and correctional services (de Vogel & Nicholls 2016). This is an area that warrants further investigation to reduce, with the aim of eradicating seclusion use and its associated trauma, for a population at risk of re‐traumatization.

Females in forensic settings often have complex histories involving childhood abuse and trauma, as well as invasive experiences of family violence, physical, and sexual abuse (e.g. Bartlett *et al*. 2014; Cooke & Bailey 2011), which may influence their experience of seclusion, particularly the exacerbation of existing trauma. Seclusion use is documented as being associated with trauma (e.g. Brophy *et al*. 2016a). A study by Hammer *et al*. (2011) found that consumers who experienced childhood abuse experienced higher rates of seclusion over time and the use of seclusion may result in re‐traumatization. This finding suggests that a female’s past experiences associated with extensive trauma maybe exacerbated during an episode of seclusion. Mental health services employ trauma‐focused interventions that aim to recognize the link between, and prevalence of childhood trauma and adverse mental health outcomes (Muskett 2014). However, the continued use of seclusion for consumers who have histories of trauma potentially undermines the importance of trauma and recovery‐focused interventions in mental health practice, particularly in a population with complex histories of trauma.

Females in forensic settings are involved in more frequent incidents of violence while admitted than males (Parkes & Freshwater 2012) and engage in more self‐harm during treatment (de Vogel & Nicholls 2016). These acts of violence towards themselves and others may be an expression of their psychological distress as a result of past experiences and trauma (Parkes & Freshwater 2012). The use of seclusion to manage violence directed towards themselves or others may further contribute to the exacerbation of trauma, instead of protecting the person or others from harm.

Seclusion reduction and eradication continue to be an important part of mental health practice. However, strategies aiming to reduce seclusion may not be effective for some consumers who present with factors associated with seclusion use, such as age, risk of violence to others, and a previous history of seclusion (Bullock *et al*. 2014). Despite seclusion reduction and elimination being a priority (Barr *et al*. 2019), seclusion remains an option to be used to manage consumer behaviour that presents a risk to themselves or to others. Consumers who are identified as being at risk of seclusion use require early identification, and effective strategies to reduce incidents of seclusion need to be implemented (Bullock *et al*. 2014). This is imperative for women, given that many women in forensic mental health settings have experienced complex trauma in their past.

The voice of consumers and certainly their experiences are required to be at the centre of seclusion reduction and eradication. This has been recently highlighted by the Royal Commission (State of Victoria 2021) which indicated that efforts to eliminate the use of seclusion in mental health settings require consumer input to understand experiences and provide alternatives to seclusion use.

Without an understanding of women’s experiences of seclusion, the psychological harm associated with seclusion will remain, and may not be able to be reduced. Given what we do know about the history of women in forensic mental health settings, and behaviours that present while admitted, the use of seclusion is problematic for this population, and puts the woman at risk of re‐traumatization. This may affect clinical and personal outcomes during and following admission. Having a better understanding of the experience of seclusion, specifically for women, may contribute to, and influence the way in which care is delivered in the context of seclusion. New understandings can evidence the development of sex or gender‐specific interventions, with less likelihood of psychological harm and re‐traumatization.

## STRENGTHS AND LIMITATIONS OF THIS REVIEW

The key strengths of this review include using a systematic search approach, clear inclusion and exclusion criteria, and initial screening of articles independently by two authors. This review is primarily limited by the small number of studies that met inclusion criteria which limit the use and generalisability of the findings; however, the themes identified may assist with the generation of new hypotheses for testing in future research. Quantitative data relating to the experience of seclusion for females compared to males was not clear in all included studies, which may have limited the ability to determine whether there are differences in the experience of seclusion for females and males. Qualitative and quantitative responses were largely not identified by sex, which restricts the scope of this review to identify differences in the experience of seclusion for males and females.

The lack of qualitative studies in this area limits the ability of consumers to have a voice about their treatment and their experiences of treatment, in this case, seclusion. The importance of understanding personal experience is crucial in the process of recovery (Jacob *et al*. 2017). There is imperative to understand consumer experiences to responsively make changes to policy, education and training, practice, and support recovery. The lived experience of consumers and their supporters (carers) has been suggested as important in understanding practices in mental health care and contributing to change (Brophy *et al*. 2016a).

## IMPLICATIONS FOR CLINICAL PRACTICE

This review provides a synthesis of what is known about the voice of consumers regarding seclusion in forensic health settings which can be used to inform future policy and practice. The consumer experience of seclusion in forensic mental health settings is not well understood and little is known about the differences in experiences of seclusion for females and males. Females and males admitted to forensic mental health settings present with differences in their experience of trauma, psychosocial, clinical, and criminological presentation, which may affect their behaviour while admitted and potentially their experience of and risk of harm from seclusion. Consideration and acknowledgement of differences between males and females are required to inform mental health policy and practice that is sensitive to sex differences, and to assist in reducing trauma associated with seclusion use, particularly for a population that is highly vulnerable. The use of seclusion in forensic mental health settings is largely perceived as a negative experience that causes harm and is used as a form of punishment. For a population who may already view a forensic setting as punitive and traumatic, the experience of seclusion may further exacerbate existing trauma and undermine therapeutic intervention. It is important to support clinicians to consider their practice with the intention of reducing, and where possible, eliminating the use of seclusion with established seclusion reduction approaches (e.g. Huckshorn, 2004; Long *et al*. 2015). Further research is required to understand the experience of seclusion for consumers as well as efforts to eliminate seclusion as a behaviour management option and inform efforts to design alternate interventions to protect consumers and others from harm.

## CONCLUSION

This integrative review found that most consumers in forensic mental health settings report seclusion to be a negative and harmful experience that negatively impacts their emotional health, and is perceived to be a punishment for their behaviour. Further research is required to understand the consumer experience of seclusion in forensic mental health settings, particularly the experience of seclusion for females. With a better understanding of the female experience of seclusion, sex or gender‐specific interventions can be developed to inform policy and support evidence to reduce and where possible eliminate the use of seclusion. With a reduction or elimination of seclusion, the harm associated with seclusion will be reduced, which will support and maintain recovery and optimize trauma‐informed care.

Seclusion continues to be available for staff to use, however, the availability of seclusion may act as a deterrent for clinicians to attempt to use other approaches to manage behaviour, in situations where seclusion would be traditionally used. Until an effective alternative approach or intervention is identified to manage a person’s behaviour, seclusion will continue to be used.

## RELEVANCE FOR CLINICAL PRACTICE

The findings of this review help clarify the consumer experience of seclusion in forensic mental health settings and highlight the need to consider consumers’ sex to understand the experience of seclusion for women in forensic mental health settings. The development and testing of sex‐specific interventions may assist mental health nurses in reducing and potentially eliminating the use of seclusion, and reducing associated trauma while supporting recovery and trauma‐informed care, and consumer outcomes.
